# Predictability of Gastric Intestinal Metaplasia by Mottled Patchy Erythema Seen on Endoscopy

**DOI:** 10.4021/gr357w

**Published:** 2011-09-20

**Authors:** Naoyoshi Nagata, Takuro Shimbo, Junichi Akiyama, Ryo Nakashima, Hyung Hun Kim, Takeichi Yoshida, Kazufusa Hoshimoto, Naomi Uemura

**Affiliations:** a Department of Gastroenterology and Hepatology, National Center for Global Health and Medicine (NCGM), Tokyo, Japan; b Department of Clinical Research and Informatics International Clinical Research Center Research Institute, NCGM, Tokyo, Japan; c Division of Internal Medicine, Kosin University College of Medicine, Busan, Korea; d Department of Gastroenterology and Hepatology, Wakayama Medical University, Wakayama, Japan; e Department of Clinical Laboratory Pathological Division, NCGM, Tokyo, Japan; f Department of Gastroenterology and Hepatology, NCGM, Kohnodai Hospital, Chiba, Japan

**Keywords:** Intestinal metaplasia, Premalignant lesion, Endoscopic finding, Erythema, White-light endoscopy, Subtype, eradication, *Helicobacter pylori*

## Abstract

**Background:**

Intestinal metaplasia (IM) is regarded as a premalignant lesion. However, endoscopic diagnosis of IM has been considered difficult. Using endoscopy, we found a unique pattern of erythema, “Mottled Patchy Erythema (MPE),” which includes severe IM. *Helicobacter pylori* (*Hp*) infection itself can cause erythema, which reflects histologic changes in the gastric mucosa. Therefore we enrolled *Hp* eradication patients to validate the relation between MPE and pathologic findings.

**Methods:**

We enrolled patients with chronic gastritis who underwent successful *Hp* eradication at least 6 months before the study. We defined MPE as multiple flat or depressed erythematous lesions. When encountering MPE on endoscopy, we performed biopsy on both the MPE site and non-MPE site. The non-MPE site was defined as an adjacent mucosa located within 3 cm of the MPE site. All biopsy specimens were evaluated immunohistochemically for IM subtype using MUC2, MUC5AC, MUC6, CD10, and CDX2 stains. The degree of IM was defined according to the Updated Sydney System. The diagnostic accuracy of the MPE findings for pathologic IM was calculated. The relation between MPE and IM subtype was also assessed.

**Results:**

A total of 102 patients were selected for the study. Of these, 55 (54%) patients had MPE. Biopsy specimens were taken from the MPE sites and non-MPE sites from these 55 patients. The IM percentages and median scores of IM were both significantly higher at the MPE sites (P < 0.001) than at the non-MPE sites. The sensitivity and specificity for MPE in the detection of histologic IM were 72.7% and 84.1%, respectively. No significant associations were observed in the expression of MUC2, MUC5AC, MUC6, CD10, and CDX2 between the MPE sites and non-MPE sites. There were no significant differences in the ratios (complete/incomplete) of IM subtypes between the two groups.

**Conclusions:**

MPE is a useful endoscopic finding to detect histologic IM without the use of chromoendoscopy and magnifying endoscopy. However, the IM subtype is difficult to identify. In the era of *Hp* eradication, MPE has the potential to become a predictive finding for the risk of gastric cancer.

## Introduction

It is believed that the development of gastric cancer involves a multi-step process, including *Helicobacter pylori* (*Hp*) infection, chronic gastritis, glandular atrophy, intestinal metaplasia (IM), and finally dysplasia [1]. IM and gastric atrophy are considered together to be risk factors for the development of intestinal-type gastric cancer and are regarded as premalignant lesions [2, 3]. Gastric atrophy can be recognized by endoscopy and correlates with histologic evaluation [4, 5]. However, the diagnosis of IM by using standard white light endoscopy has been considered to be difficult due to IM lacking distinction in color and its presence as multiple flat lesions [6, 7].

Recently, we found that a unique erythematous finding on endoscopy could be observed even after *Hp* eradication [8]. We describe this finding as “Mottled Patchy Erythema (MPE).” MPE can be recognized as multiple flat or slightly depressed erythematous lesions under standard white light endoscopy; pathologically, it includes severe IM [8]. *Hp* infection itself can cause erythema, seen on endoscopy, which reflects histologic changes such as infiltration of inflammatory cells and edema [9]. Therefore, we enrolled patients who underwent *Hp* eradication in order to validate the relation between MPE and pathologic findings.

## Methods

### Patient selection

Patients with chronic gastritis who underwent successful *Hp* eradication at least 6 months prior to the study were prospectively enrolled for the study at the National Center for Global Health and Medicine (NCGM) between January 2008 and December 2008. Exclusion criteria included the use of non-steroidal anti-inflammatory drugs (NSAIDs), antacids, and anti-thrombotic drugs during the 4 weeks before endoscopy. We also excluded patients with a history of gastric surgery, hemorrhagic disease, liver cirrhosis, renal failure, heart failure, and early or advanced gastric cancer. Written informed consent was obtained from participants in accordance with the Declaration of Helsinki and its subsequent revision. The study protocol was approved by the Ethics Committee of the NCGM.

### *Helicobacter pylori* eradication

Patients with chronic gastritis and peptic ulcer disease induced by *Hp* infection underwent eradication therapy. Patients were treated with a 7-day regimen consisting of amoxicillin, clarithromycin and a proton pump inhibitor (PPI) twice daily, which was the standard first-line regimen approved in Japan. If eradication was not successful, a second regimen consisting of amoxicillin, metronidazole, and PPI was administered. Eradication was confirmed by negative histologic examination of the gastric biopsies, together with a negative 13C-urea breath test (13C-UBT) 2 to 3 months after the completion of eradication therapy. When all of the tests were negative, a patient was defined as negative for *Hp* infection.

### Endoscopic assessment

**Figure 1:**
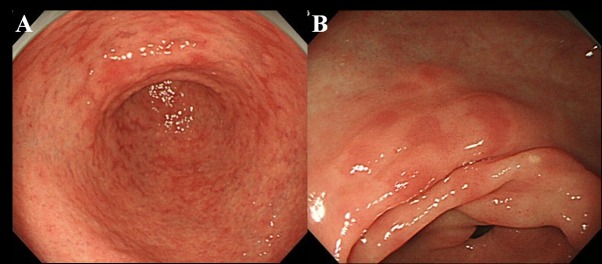
Endoscopic finding of MPE. (A) Multiple and flat erythema in the antrum; (B) Flat and depressed erythema in the lesser curvature of the antrum.

At least 6 months after the eradication of *Hp*, patients underwent endoscopic examination. We used a high resolution endoscope without magnification (Olympus videoscope, model GIF-H260) to observe the presence of MPE in the gastric mucosa. We defined MPE as multiple flat or slightly depressed erythematous lesions that were distinguishable from congested mucosa, hemorrhage, angioectasia, spotty erythema, and linear erythema (Fig. 1A, B). We also distinguished between MPE and reddish mucosa with a regenerating epithelium accompanied by ulcer or ulcer scar.

**Figure 2:**
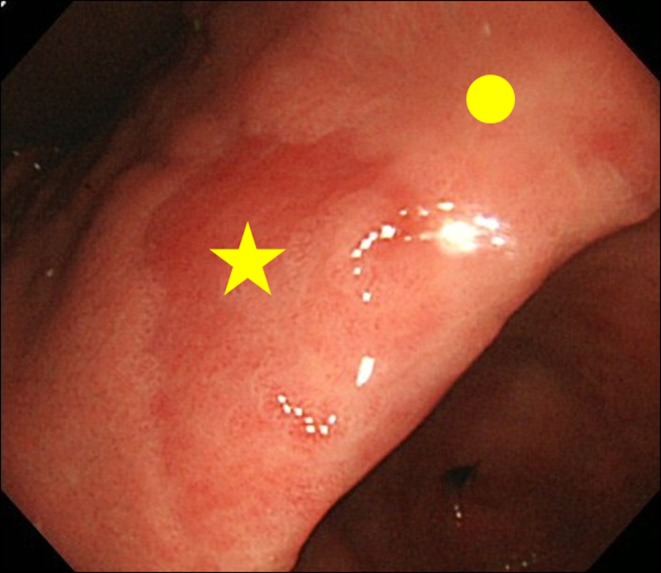
Biopsy site. (★) Biopsy site of MPE; (•) Biopsy site of non-MPE. Non-MPE site was defined as an adjacent mucosa located within 3 cm of MPE site.

When encountering a finding of MPE finding at endoscopy, we performed biopsy of both the MPE site and the non-MPE site. The non-MPE site was defined as an adjacent mucosa located within 3 cm of the MPE site (Fig. 2).

The extent of the atrophic border was classified into 3 stages: mild, moderate and severe, as defined by Kimura and Takemoto [4].

### Histologic assessment

**Figure 3:**
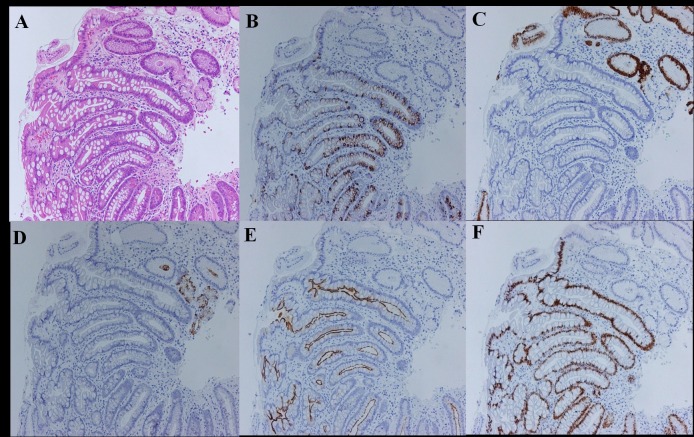
Evaluation for intestinal metaplasia using immunohistochemical staining (complete type case). (A) Hematoxylin and eosin stain; (B) MUC2 stain; (C) MUC5AC stain; (D) MUC6 stain; (E) CD10 stain; (F) CDX2 stain.

Biopsy specimens were sent to our laboratory, fixed in 10% buffered formalin. The specimens were processed, embedded in paraffin, and cut into 4 µm sections. Slides from each specimen were stained using hematoxylin-eosin (HE) (Fig. 3A) and immunohistochemical staining. The immunohistochemical stains included the following: (1) MUC2, a marker of intestinal mucin that is useful for detecting goblet cells (Fig. 3B), (2) MUC5AC, a marker of gastric mucin, which is expressed in mucous neck cells and the foveolar epithelium (Fig. 3C), (3) MUC6, a marker for gastric mucin, which is expressed in mucous cells of the neck zone of the body and pyloric glands of the antrum (Fig. 3D), (4) CD10, a useful marker for detecting the brush border of the small intestine (Fig. 3E), and (5) CDX2, a marker for the presence of IM (Fig. 3F) [10-14]. We used CDX2 because recent studies have shown that CDX2 expression could be observed extensively in IM glands, and did not disappear after eradication of *Hp* [15, 16]. The presence of IM was confirmed by both HE and immunohistochemical staining.

The subtypes of IM were classified as complete or incomplete types. The complete type was defined as decreased expression of gastric mucin (MUC5AC or MUC6) and co-expression of intestinal mucin (MUC2) and CD10 (Fig. 3A-F). The incomplete type was defined as the expression of gastric mucin (MUC5AC or MUC6) and MUC2. Because no established criteria exist to categorize a case as having both the complete and incomplete types in one section, we assigned the IM subtypes as the prevalent type [15, 16].

The degree of IM was scored based on the Updated Sydney System (0: none, 1: mild, 2: moderate, 3: marked) [9].

### Statistical analysis

We divided the patients into two groups based on whether the patients were positive or negative for MPE. We used Student's t-test to compare age and period of eradication. The chi-square test or Fisher’s exact probability test were used for the sex ratios and the degree of endoscopic atrophy in the two groups.

We also compared the histologic findings between the MPE sites and the non-MPE sites. To compare the differences between the two biopsy sites, we used the Wilcoxon Matched-Pairs Signed-Ranks Test for the IM median score, and Fisher’s exact test for the prevalence of IM, IM subtypes and IM phenotypes. The sensitivity, specificity, positive and negative predictive values, and positive and negative likelihood ratios of MPE seen on endoscopy for the detection of pathologic IM were calculated. P values < 0.05 were considered significant. All statistical analyses were performed with Stata software, version 10 (StataCorp LP, College Station, TX, USA).

## Results

### Patient characteristics

During the study period, 157 patients who underwent upper endoscopy and received *Hp* eradication were reviewed. We excluded 52 of the 157 patients from analysis for any of the following criteria: use of antacids (31), anti-thrombotic drugs (17), or NSAIDs (3); or history of liver cirrhosis (5), heart failure (1), or early gastric cancer (2). More than one exclusion criterion applied to some patients.

A total of 102 patients were therefore selected for analysis after exclusion. Of these, there were 55 (54%) patients with MPE. No significant differences were noted in the mean age and sex between MPE-positive and -negative patients (Table 1). There were no significant differences between the two groups after the *Hp* eradication period. However, gastric atrophy with a higher severity (moderate to severe) appeared significantly more frequently in the MPE-positive group compared with the MPE-negative group (positive 65.6% versus negative 45.0%, P = 0.035).

**Table 1:** Demographic Characteristics of Patients (n = 102)

	MPE + (n = 55)	MPE - (n = 47)	P value
Mean age ± SD (years)	66.1 (14.0)	62.1 (14.4)	0.080
Male sex	33 (60.0%)	22 (46.8%)	0.183
Period after eradication ± SD (months)	27.8 (23.8)	23.4 (13.7)	0.134
Endoscopic gastric atrophy (moderate to severe)	36 (65.5%)	21 (45%)	0.035

### Endoscopic findings and pathologic features

The percentage of IM at the MPE sites was significantly higher than at the non-MPE sites (MPE 87.3% versus non-MPE 32.7%, P < 0.001) (Table 2). The median IM score was significantly higher at the MPE sites than at the non-MPE sites (MPE 2 versus non-MPE 0, P < 0.001) (Table 2).

**Table 2:** Comparison of IM Score Between MPE and Non-MPE Site (n = 55)

IM score	MPE site (n = 55)	Non-MPE site (n = 55)	P value
0	7 (13%)	37 (67%)	
1	15 (27%)	9 (16%)	
2	16 (29%)	5 (9%)	
3	17 (31%)	4 (7%)	< 0.001

The sensitivity and specificity of MPE in the detection of pathologic IM were 72.7% (95% CI: 59.0 to 83.9) and 84.1% (95% CI: 71.2 to 92.2), respectively (Table 2). The positive predictive value, negative predictive value, positive likelihood ratio, and negative likelihood ratio were 87.3% (95% CI: 75.5 to 94.7), 67.3 % (95% CI: 53.3 to 79.3), 4.57 (95% CI: 3.62 to 5.62), and 0.32 (95% CI: 0.21 to 0.47), respectively (Table 2).

No significant associations were observed in the expression of MUC2, MUC5AC, MUC6, CD10, and CDX2 between the MPE sites and the non-MPE sites (Table 3). There was no significant difference in the ratios (complete/incomplete) of IM subtypes between the two groups (MPE: 18/30 versus non-MPE: 8/10, P = 0.778).

**Table 3:** Phenotypes and Subtypes of Intestinal Metaplasia

	MPE site (n = 48)	Non-MPE site (n = 18)	P value
Phenotypes			
MUC2 positive	48 (100%)	18 (100%)	
MUC5AC positive	28 (58.3%)	9 (50.0%)	0.587
MUC6 positive	11 (22.9%)	6 (33.3%)	0.528
CD10 positive	36 (75.0%)	14 (77.8%)	1.000
CDX2 positive	41 (85.4%)	15 (83.3%)	1.000
Subtypes			
Incomplete/Complete	18/30	8/10	0.778

## Discussion

In this study, we focused on a unique erythematous appearance seen on endoscopy after *Hp* eradication. We called this finding “MPE” and we found that the presence of MPE as seen on endoscopy was typically characteristic of pathologic IM.

The diagnosis of IM with conventional endoscopy has been considered difficult because IM usually appears in flat mucosa and exhibits few morphologic changes. Kaminishi et al reported “ash-colored nodular change” as an indicator for IM; the accuracy of these investigators’ findings was high, with a specificity of 98-99%, but the sensitivity was low (6-12%). Kaminishi et al noted that conventional endoscopy is less useful for confirming the diagnosis of IM [5]. Recent studies have emerged concerning the endoscopic finding of IM using magnifying endoscopy. It has been reported that the distinctive findings of gastric pits seen with methylene blue chromoendoscopy and the “villus-like appearance” seen with confocal endoscopy have been useful for diagnosing IM [17-19]. Uedo et al reported that the appearance of “a light blue crest” (LBC) is an accurate sign for the presence of IM, as seen with narrow band imaging (NBI)-magnifying endoscopy [20]. However, due to the high equipment costs and the additional skills and time required for closer examination using such special tools as NBI or magnifying endoscopy, screening with this equipment is not practical in daily clinical practice [21]. In addition, there is an increased risk of damage to the DNA in the gastrointestinal mucosa when using chromoendoscopy with methylene blue followed by white light, requiring caution in its use [22]. Therefore, it is more beneficial to diagnose IM by finding MPE without the use of chromoendoscopy and magnifying endoscopy. Our study results suggest that the presence of IM can be diagnosed with standard endoscopy without biopsy.

Why can MPE be observed even after the eradication of *Hp?* This could be attributable to the histologic changes within the gastric mucosa. The remarkable histological changes following eradication include improvements in the infiltration of inflammatory cells, epithelial hyperplasia and edema [23, 24]. The endoscopic images of erythematous and edematous mucosa that appear to be improved reflect these histologic changes. Therefore, we speculate that MPE consists of a remaining area of persistent erythematous IM and a rapidly recovered non-IM area resulting from successful *Hp* eradication. The eradication of *Hp* caused the contrast between the MPE area and non-MPE areas to become clearer. However, it is unknown why only the MPE site is observed as an erythematous mucosa. It can probably be inferred that a highly dense area of microvessels surrounds the metaplastic glands; this has not been elucidated in this study. Additionally, the IM score at the MPE site was significantly higher than the score at the non-MPE site. We speculate that the appearance of erythematous mucosa is associated with the presence of many metaplastic glands.

The present study demonstrated that the IM complete subtype was predominantly found in the gastric mucosa. The subtypes of IM have been classified into either the complete or incomplete type; these are the most widely used subtypes [25]. Several studies have shown that the complete type does not exhibit any increased risk for developing carcinoma, whereas the incomplete type is associated with an increased risk of malignant transformation [26, 27]. However, the association between the subtypes and the risk of gastric cancer is not widely accepted [28]. At present, it is difficult to identify either of the subtypes using standard endoscopy.

It has been reported that *Hp* eradication therapy is effective in preventing both gastrointestinal ulcer as well as the development of gastric cancer [29]. These uses of *Hp* eradication therapy will likely emerge in clinical practice in the near future. However, caution should be taken against the risk of the development of gastric cancer after *Hp* eradication. The characteristics of gastric cancer after eradication have been reported to include pathologically severe IM at the corpus and severe gastric atrophy as detected on endoscopy [30]. Therefore, it is necessary to carefully observe the presence of IM even after *Hp* eradication.

In conclusion, the presence of MPE on endoscopic examination is characteristic of pathologic IM. It would be beneficial in clinical practice to be able to diagnose pathologic IM without chromoendoscopy or magnifying endoscopy. MPE has the potential to become a predictive finding for the risk of gastric cancer in the era of *Hp* eradication.
